# Undernutrition among the children below five years of age in Uganda: a spatial analysis approach

**DOI:** 10.1186/s12889-023-15214-9

**Published:** 2023-02-24

**Authors:** Vallence Ngabo Maniragaba, Leonard K. Atuhaire, Pierre Claver Rutayisire

**Affiliations:** 1 African Centre of Excellence in Data Science, University of Rwanda, Kigali, Rwanda; 2 College of Business and Management Sciences, Makerere University, Kampala, Uganda

**Keywords:** Undernutrition, Under-five children, Spatial analysis, Hot spots, Cold spots, Uganda

## Abstract

**Background:**

Undernutrition is a health condition caused by a lack of enough food intake, not having enough of the right combination of food nutrients, or the body’s failure to utilize the food eaten resulting in either, stunting, being underweight, or wasting. Globally, undernutrition affects more than 149 million under-five children, while in Uganda about 3 in every 10 children suffer from undernutrition. Undernutrition and its risk factors among under-five children in Uganda were unevenly distributed across the country and a study that focused on spatial distribution was prudent to examine the nature of the problem and salient factors associated with it. The current study addressed the issues of spatial heterogeneity of undernutrition and its determinants with the goal to identify hot spots and advise policymakers on the best actions to be taken to address the problem.

**Methods:**

Data were obtained from the 2016 Uganda Demographic and Health Survey. Prevalence rates and percentages of risk factors were combined with the Uganda district shape file to allow spatial analysis. Moran’s I, Getis-Ord (GI*), and Geographically Weighted Regressions were respectively used to establish the local, global, and geographically weighted regressions across the country. Stata 15 and ArcGIS 10. 7 soft wares were used.

**Results:**

The results indicate that undernutrition in Uganda shows varies spatially across regions. Evidence of hot spots exists in the Karamoja and Arua regions, cold spot areas exist around the central part of the country while the greatest part of Western Uganda, Northern, and Eastern were not significant.

**Conclusion:**

The study reveals that a variation in the distribution of undernutrition throughout the country. Significant spatial patterns associated with undernutrition as identified through the hotspot and cold spot analysis do exist in Uganda. Programs targeting to reduce the undernutrition of under-five children in Uganda should consider the spatial distribution of undernutrition and its determinants whereby priority should be given to hotspot areas. The spatial intensity of undernutrition and its determinants indicate that focus should be tailored to meet the local needs as opposed to a holistic national approach.

## Introduction

Undernutrition, also known as malnourishment, is a health condition which is caused by a lack of enough food intake, not eating enough of the right combination of food, or the body’s failure to utilize the food eaten. It manifests itself in two forms: undernutrition or over nutrition. Deficiency of food nutrients results in stunting, being underweight or body wasting. The 2020 global statistics indicate that about 149 million children under 5 years of age were estimated to be stunted (too short for their age), 45.4 million were estimated to be wasted (too thin for their height) and about 38.9 million children under 5 years of age are overweight [1]. Undernutrition makes children much more vulnerable to diseases and even death. Globally, close to 45.4 million of all death among children under 5 years of age are linked to undernutrition and many of the victims are in developing countries [1]. The medical, social, economic, and developmental effects of the global undernutrition burden are lasting and serious for individuals and their families, communities and countries due to its complex and dynamic nature [2].

The burden and developmental effects of undernutrition are not uniform world over. Countries with greater prevalence are faced with greater burden as well. For example, of the 144 million of the world’s stunted under-five children, about 94% are within Asia and Africa alone while only about 6% are distributed within the rest of the regions of the globe.

Asia alone accounts for about 54% while African accounts for about 40% of the worlds’ stunted under-five children. A similar scenario of un-even distribution of children below 5years of age suffering from body wasting was observed whereby about 96% of all the world’s wasted children were in Asia and Africa alone with Asia accounting for about 69% while African accounted for about 27% [1].

Subsequently, the distribution of the 59.5 million children below five years of age who are stunted in Africa is not uniform at all. With about 23.1m or ~39% prevalence, East Africa has the highest prevalence in Africa, followed by West Africa with about 17.8m or about 30% prevalence rate. The lowest rates of stunting are in Southern and Northern Africa respectively. Within East Africa, about 4 in every 10 children or about 35% are stunted while about 3.5% of children below five years of age faced with the problem of body wasting.

Uganda is among the countries in East Africa with high levels of undernutrition whereby about 29 percent or 3 in 10 children below 5 years of age are stunted while about 3.5% of all children below 5 years of age in Uganda are faced with body wasting [1].

A sample of 4,530 children aged between 0-59 months had their anthropometric measurement successfully captured during the sixth Uganda Demographic and Healthy Survey (UDHS) of the year 2016. Based on this sample, the prevalence of undernutrition was calculated for all regions in Uganda with an aim to identify distribution and variation in undernutrition prevalence in Uganda (Table 1). The established prevalence shows great variations from region to region with the highest prevalence of stunting among the under-five children being recorded in Toro region (Western part of Uganda), the lowest stunting rates were recorded in regions of Teso (Eastern part of Uganda), while Arua region had the highest levels of wasting (Table 1).

**Table 1:** Prevalence rates of undernutrition for selected regions in Uganda

Region	Stunted (%)	Body-wasting (%)	n
Toro	40.6	3.4	375
West Nile	39.0	6.2	319
Bugisu	36.0	5.0	217
Teso	14.3	2.2	369
Kampala	18.4	3.9	176
**National level**	**30.9**	**4.1**	**4,530**

From Table 1, as indicated by the prevalence rate, it can be noted that, children under-five years of age in different regions of Uganda have different risks of being undernourished. Interventions aimed at addressing the problem would be best tailored to the local needs after spatially examining the problem and the spatially based influencers of the problem. The inequalities in the undernutrition distribution across regions in Uganda calls for an approach that establishes the core reasons behind observed phenomenon region by region. Spatial analysis also known as the “Science of Where” is best fit for this purpose. The “Science of where” is an important breakthrough in understanding the undernutrition problem across the country. It helps in establishing the hot spots and cold spots as well as the risk factors behind the observed status quo.

Spatial analysis which refers to statistical analysis based on patterns and underlying processes across a given region is thus the best-fit approach for understanding the under-five children’s undernutrition phenomenon in Uganda. Spatial analysis is a kind of geographical analysis that elucidates patterns of phenomenal characteristics and spatial appearances in terms of geo-statistics and geometrics as well as their locations [3]. Evidence of high spatial clustering on the provision of basic services, per capita expenditure as well as expenditure on food though the magnitude of the spatial clustering varied from city to city, while other indicators considered showed little evidence of spatial clustering was realised in seven different cities in Africa, Asia, and Latin America [4].

Various studies done on spatial analysis of malnutrition in Uganda and other areas employed various methods which include; ArcGIS Getis-Ord Gi* statistic [5], Multilevel and spatial analysis, spatial regression methods and multi-scale geographically weight regression (MGWR) [2], Bayesian geo-statistical Weibull proportional hazards models [6], geo-additive semi-parametric mixed model and Markov Chain Monte Carlo techniques [3], multi-level multivariable logistic regressions and geospatial variations [4] and Bayesian framework [7]. The current study examined the occurrences of both the global and local clustering of the undernutrition problem using the ArcGIS Getis-Ord Gi* and the Moran’s I statistics, and the dependences or independences between undernutrition and its factors using grouping analysis and GWR.

The current study banked on spatial analytical techniques to examine how the spatially related risk factors influence undernutrition of children below five years of age in Uganda. With this approach, the researchers were able to flexibly determine to what extent the spatial pattern of undernutrition is depend on detectable spatially located factors in different regions of the country.

### Data and data source

Secondary data from Demographic and Health Surveys (DHS) programme was combined with the district shape files for Uganda (Uganda District shape file, 2014). The Uganda Demographic and Health Survey (UDHS) data for this study was accessed with permission from the DHS website : https://dhsprogram.com/data/dataset_admin/index.cfm. Data for UDHS 2016 was then downloaded from the database as a whole, thereafter, the variables most relevant for this study were extracted for use. The most important variables such as anthropometric variables, and relevant risk factors were extracted from the children’s records (KR) file from UDHS (2016) database. The extracted and modified variables were then merged with the 2016 district shape file to enable spatial analysis of undernutrition and its determinants amongst the under-five children across the country.

The UDHS design and data collection process is carried out by a highly trained team and can thus makes the data credible. Respondents are selected from all the regions of Uganda and this makes it representative. Normally UDHS design is benchmarked on the Uganda National Population and Housing censuses (NPHC). Specifically, the sampling frame for the 2016 UDHS was the frame for the Uganda National Population and Housing Census (NPHC) that had been conducted in 2014 implying that the UDHS Enumeration Areas (EAs) were extracted from the Uganda Population and Housing Census (UPHC) EAs. UDHS (2016)’s design relied on a multi-stage stratified random sampling technique, a technique through which 696 EAs were selected from the 2014 Uganda NPHC, with, 162 EAs in urban areas while 535 were rural based EAs. In total, a representative sample of 20,880 households (30 per EA) were randomly surveyed and interviewed for the 2016 UDHS. From the 20,880 selected households,18,506 women were successfully interviewed. The survey was conducted from all the 15 regions of Uganda inclusive of Kalangala Island. Data was collected from all the 112 districts that formed the third administrative unit in the country by the time of UDHS 2016. The type of sampling technique used, being of one of the probability sampling techniques, makes the approach of spatial statistical analysis possible and more appropriate for this study. More information about the sampling and survey execution can be accessed from the UDHS report [6].

Scope wise, children have no capacity to consent and therefore there caretakers especially mothers provided most of the information. All women aged 15-49 who were either permanent residents of the selected households or visitors who stayed in the household the night before the survey were eligible to be interviewed. Height and weight information was also collected from eligible women and men, as well as children age 0-59 months. Anthropometric measurements were successively recorded for a total of 4,530 children (0-5years) of age. This study thus uses data for 4,530 children whose records of age, height and weight are complete and this sample size is statistically sufficient for spatial analysis. The 15 regions captured in the (UDHS, 2016) were not statistically sufficient to guarantee spatial analysis and this necessitated the use of district shapefile instead of regions.

### Data analysis

Data analysis started with exploratory spatial data analysis (ESDA). The ESDA helped in establishing spatial descriptive statistics especially autocorrelation and then the identification of the clusters, cold and hotspot areas of under-nutrition across the country.. Theoretically, exploratory spatial data analysis (ESDA) is the extension of exploratory data analysis (EDA) to the problem of detecting spatial properties of data sets where, for each attribute value, there is a locational datum [8]. The locational datum references the point or the area to which the attribute refers. Spatial patterns of variables of prevalence of malnutrition as well as the subsequent regression models were evaluated using the ArcGIS version 10.

Accordingly, in this study, the researchers examined the possibilities of spatial patterns, spatial clusters, spatial autocorrelation, spatial wild characters or outliers, and spatial heterogeneity of undernutrition across the country. Spatial autocorrelation which is also known as spatial dependence is defined as the similarity or dissimilarity measure between two values of an attribute that are spatially close or the description of presence (or absence) of variations in a variable. Positive spatial autocorrelation implies that high or low values of an attribute tend to cluster in the same locality, while, negative spatial autocorrelation implies that high vales of undernutrition tend to be surrounded by low values of undernutrition in the neighborhood and low values of undernutrition tend to be surrounded by high values of undernutrition in the neighborhood. Spatial patterns of undernutrition were measured by spatial statistical indices particularly the Moran’s I and the Getis-Ord (Gi*) indices. Spatial grouping approach was used to examine factors that were associated with undernutrition across the country while the cause-and-effect regression analysis was done using Geographically Weighted Regressions (GWR), a tool included in ArcGIS 10.7.

### The Moran’s I

Moran’s I statistic was vital in establishing the association between the variable values at a single location with those at other locations [9, 10]. Moran’s I statistic, takes into account both the value and location of a variable, and it can reveal important information about the dependence or independence of the variable from the surroundings as expressed in Eq. 1.

1 $$I =\frac{N{\sum }_{i}{\sum }_{j}{w}_{ij}({X}_{i}-\overline{X })({X}_{j}-\overline{X })}{({\sum }_{i}{\sum }_{j}{w}_{ij})({X}_{j}-\overline{X }{)}^{2}}$$

where, N is the number of cases, $X_{i}$ is the variable prevalence of malnutrition at a certain prevalence of malnutrition, and $w_{ij}$ is the weight used for the comparisons made between locations $_{i}$ and $j$. $w_{ij}$ is a weighted matrix based on distance, and also is the reversed distance between locations $_{i}$ and $j$. As far as this study was concerned, sampling weights (v005) was used for the comparison and harmonization of the undernutrition prevalence between different locations. In such a way, locations with more samples are given more importance than locations with fewer samples.

### The Getis-ord (Gi*) statistic

In this study, it was vital to identify spatial patterns in undernutrition and its determinants across the country. This approach was key in identifying areas with either significantly high or low intensity of the undernutrition problem in Uganda. This was accomplished using the Getis-Ord local statistics given as;

2 $${G}_{I}^{*}=\frac{ {\sum }_{j=1}^{n}{w}_{i,j}{x}_{j}-\overline{X}{\sum }_{j=1}^{n}{w}_{i,j}}{S{\sqrt{\frac{[n\sum_{j=1}^{n}{{w}^{2}}_{i,j}-(\sum {w}_{i,j}{)}^{2}]}{n-1}}}}$$

where.

Xi:the prevalence of undernutrition at location i.

Xj:the prevalence of undernutrition at location j.

Wij:the elements of weight matrix.

n:the number ofobservations

3 $$\overline{X }=\sum_{j=1}^{n}\frac{{x}_{j}}{n}$$

4 $$\mathrm{S}=\sqrt{\sum_{j=1}^{n}\frac{{x}_{j}^{2}}{n}}-{\left(\overline{X }\right)}^{2}$$

Originally developed by Getis [5] and Ord [11], Gi* statistic (Eq. 2) was used to study the existence of identifiable spatial patterns. The general Gi* is global in nature in that the overall degree of spatial interdependency is studied resulting in a single index for the entire study area. Just like Moran’s I, the global statistics are usually too general in a way that local patterns are likely to appear homogeneous and hard to detect. The fact that the level of spatial dependency may vary significantly across the space suggests that the capacity to detect and pinpoint spatial heterogeneity is more desirable [12]. It therefore became apparent to develop, the local Moran’s I by decomposing global Moran’s I to compensate for such limitations. The family of Moran indices, still, did not have the ability to discriminate between hot spots and cold spots areas. The Gi* index therefore came in handy to solve the problem of identifying hotspots and cold spots.

The expected Getis-ord (G) for a threshold distance, d, is defined as:

5 $$E\left[G(d)\right]=\frac{W}{n\left(n-1\right)}$$

where $W$ is the sum of weights for all pairs of locations $W={\Sigma }_{i}{\Sigma }_{j}{W}_{ij}$. Assuming normal distribution, the variance of $G$ and the $\mathrm{Z}$ statistic are defined as:

6 $$Var\left[G\left(d\right)\right]=E\left({G}^{2}\right)-{E}^{2}\left(G\right)$$

with a standard error

7 $$SE\left[G\left(d\right)\right]=\sqrt{\mathrm{var}\left[G\left(d\right)\right]},$$

thus

8 $$Z\left[G\left(d\right)\right]=\frac{G\left(d\right)-E\left[G\left(d\right)\right]}{S.E.\left[G\left(d\right)\right]}$$

To identify the spatial deployment patterns, we estimated the mean center and the standard distance. The mean center in as used in this study is the latitude and longitude coordinates of all features within the study scope, and its calculation is proper for tracking both the changes that happened in the spatial distribution of features and their associations. The mean center is calculated as follows;

9 $$\overline{X }=\sum_{i=1}^{n}\frac{{x}_{i}}{N}$$

10 $$\overline{Y }=\sum_{i=1}^{n}\frac{{y}_{i}}{N}$$

$$xi and yi$$ are the coordinates of the feature $$i$$ while $$N$$ is total number of features. The standard distance is a statistic that measures the degree to which features are concentrated or dispersed around the geometric mean center. $$SD$$ Statistic can be estimated as follows:

11 $$SD=\sqrt{\frac{\sum ({x}_{i}-\overline{X }{)}^{2}}{n}+\frac{\sum ({y}_{i}-\overline{Y }{)}^{2}}{n}}$$

### Modelling the spatial effects: the geographically weighted regression (GWR) analysis

The modelling approach has the power to deal with spatial non-stationarity especially since the mean values vary by different locations across the country. The approach has been used widely in similar cases where determining the influence of risk factors at local level were the goals. Conventionally, the GWR is illustrated as summarized in Eq. 1 below

12 $$Yi={\sum }_{j=1}^{p}Xij\beta ij+Ei$$

*Y*_*i*_ denotes the value of the dependent variable at location *i*,

*X*_*ij*_ denotes the value of the j^*t*h^ independent variable for location *i*,

*β*_*ij*_ denotes a location-specific coefficient corresponding to *X*_*ij*_,

*ɛ*_*i*_ denotes a random error at location *i*.

and the local coefficients of the model are estimated as in Eq. *2*

13 $$\widehat{B\beta }={\left({X}^{T}{G}_{i}X\right)}^{1}{X}^{T}{G}_{i}Y$$

where **G**_*i*_ is a matrix of location-specific weights. This weights matrix is a diagonal matrix whose diagonal elements, (*g*_*i*1_, *g*_*i*2_, *g*_*i*3_, …, *g*_*in*_), represent weights for each observation used to estimate a local parameter at its location *i*. The sampling weights were calculated base on the total samples (v005) selected from each location *i*.

## Results

A step-by-step process adopted in carrying out an exploratory spatial data analysis (EDA).

Through EDA, the researchers were able to visualize the main spatial distributions and outliers, patterns of spatial associations (spatial dependencies or independencies), cluster, hot spots as well as cold spot areas for undernutrition. Spatial econometric models that incorporated spatial effects were generated in order to provide a deeper understanding of the spatially related risk factors of undernutrition amongst the under-five children in Uganda. Thus far, the correlation between different exogenous variables and the prevalence of undernutrition was analyzed. Later on, spatial maps that indicated either hotspots or cold spots of undernutrition and many other variables of interest were carried out. Finally, EDA opened a way to spatial econometric regression modeling.

### Exploratory data analysis for undernutrition amongst the under-five children in Uganda

**Fig. 1:**
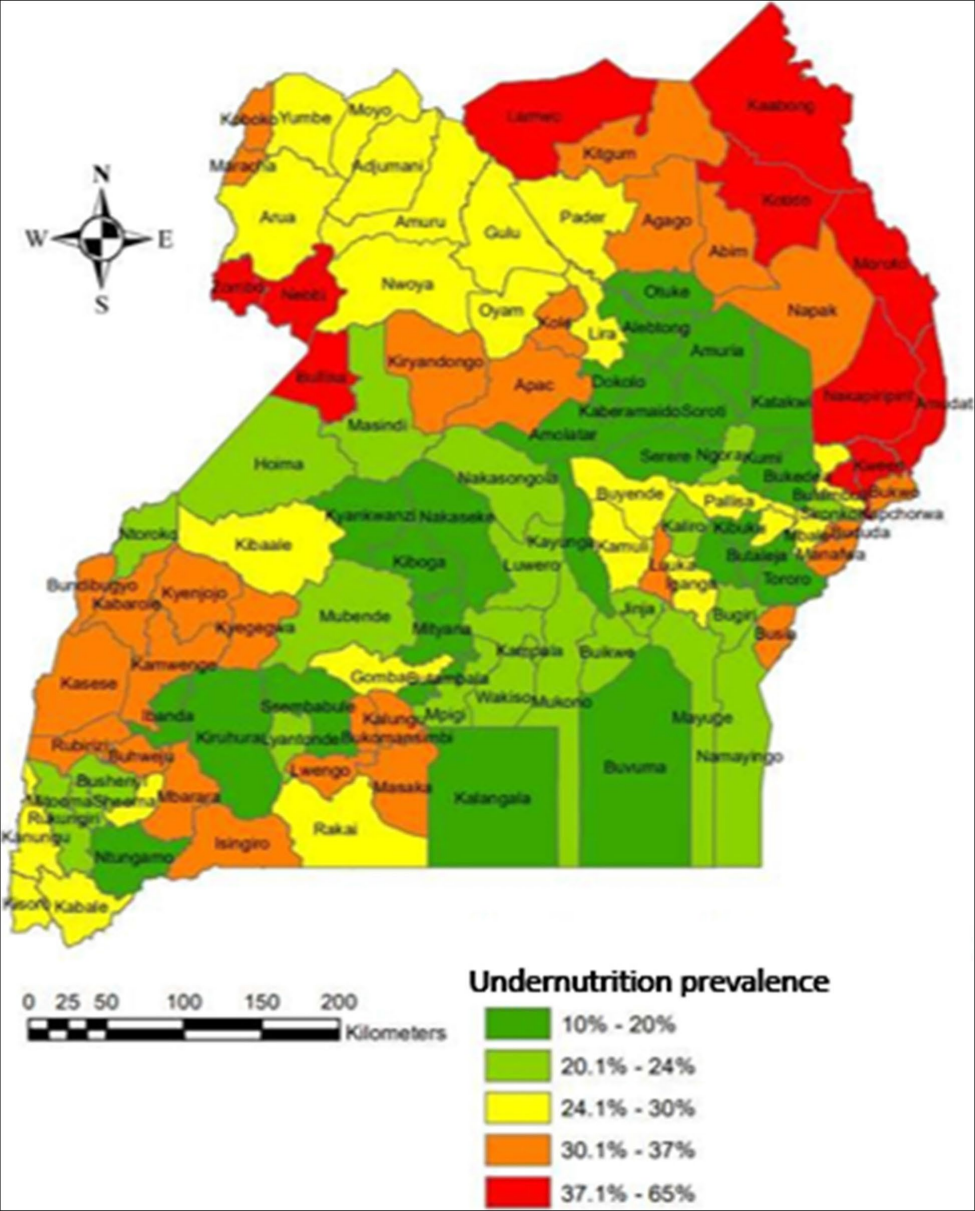
The spatial variations of undernutrition of under-five children in Uganda

**Fig. 2:**
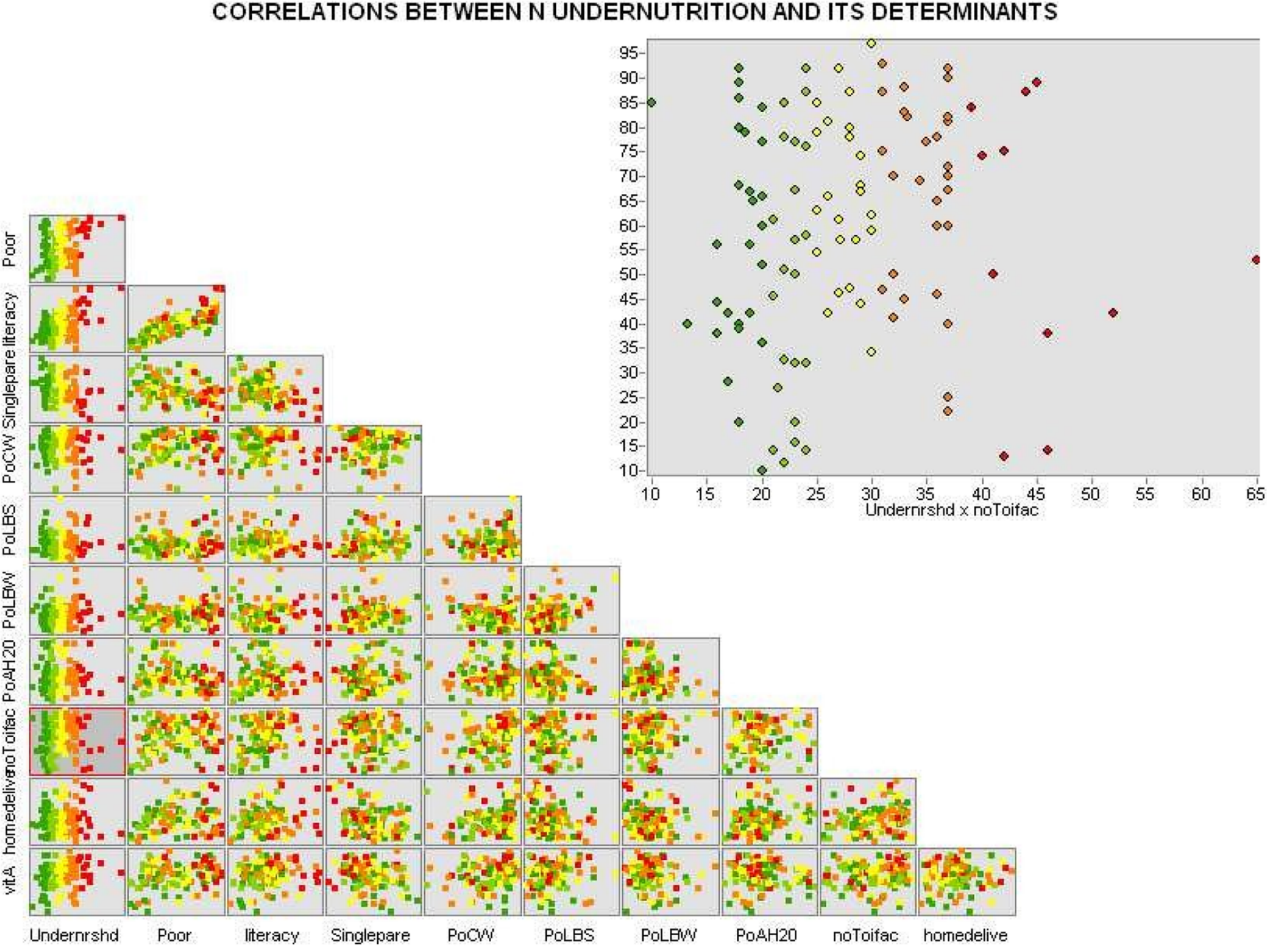
Spatial autocorrelation between undernutrition and its determinants whereby; vitA (percentage of children who had got vitamin A), homedelive (percentage of home deliveries), noToiletfac (percentage of households with no toilet facilities), PoH2O (percentage of households with access to safe drinking water), PoLBW (percentage of low birth weight children), PoLBS (percentage of low birth size children), PoCW (percentage of mothers who were working at the time of interview), Singlepare (percentage of single parents), literacy (percentage of mothers who were literate), Poor (percentage of households under wealth quintile 1 and 2 and Undernrshd (Percentage of under-five children who were undernourished)

Exploratory data analysis involved the preliminary analysis that was carried out to identify major attributes that within the data of under-five children’ undernutrition status whereby the focus was on undernutrition as a response variable. After exploratory analysis, hotspot analysis, cluster analysis and spatial econometric modeling followed suite. Under this section, spatial analysis maps (Fig. 1) and spatial autocorrelation graphs specifically scatter diagrams (Fig. 2), were generated and analyzed. Variables considered at this level of analysis were; the percentage of undernourished children by district, literacy rate which was proxied by percentage of parents who were able to read and write, water accessibility, percentage of children with low birth weight, percentage of children with small size at birth, percentage of households with no toilet facilities, mother’s working status, percentage of households within the low and the lowest wealth quintiles, percentage of deliveries at health centers and percentage of households with single parents.

From the map in Fig. 1, it was observed that undernutrition for children below five years of age in Uganda was spatially but not evenly distributed throughout the country. It is further observed that the highest prevalence of under-five children undernutrition exists within the Karamoja region of North Eastern Uganda. some few pockets in the North-Western and Northern parts of the country. Specifically, Karamoja region was followed by the Toro region in having the highest under-five undernutrition. Within Ankole region, Mbarara and Buhweju and Isingiro districts had the highest prevalence rates of under-fives under-nutrition. Bundibugyo had the highest prevalence within the Toro region while Masaka had the highest prevalence within the South-Buganda region. The lowest prevalence of under-five children undernutrition centers around the central region of the country as indicated by the legend on the map. It was also observed that some good proportion of Uganda had less than 25% prevalence of under-five children undernutrition.

Spatial autocorrelation analysis was carried out to establish the possibility of spatial correlation between the variables believed to explain the deviances in the spatial distribution of undernutrition. Overall, the output indicated some strong correlation between most variables and undernutrition prevalence as indicated in Fig. 2.

**Fig. 3:**
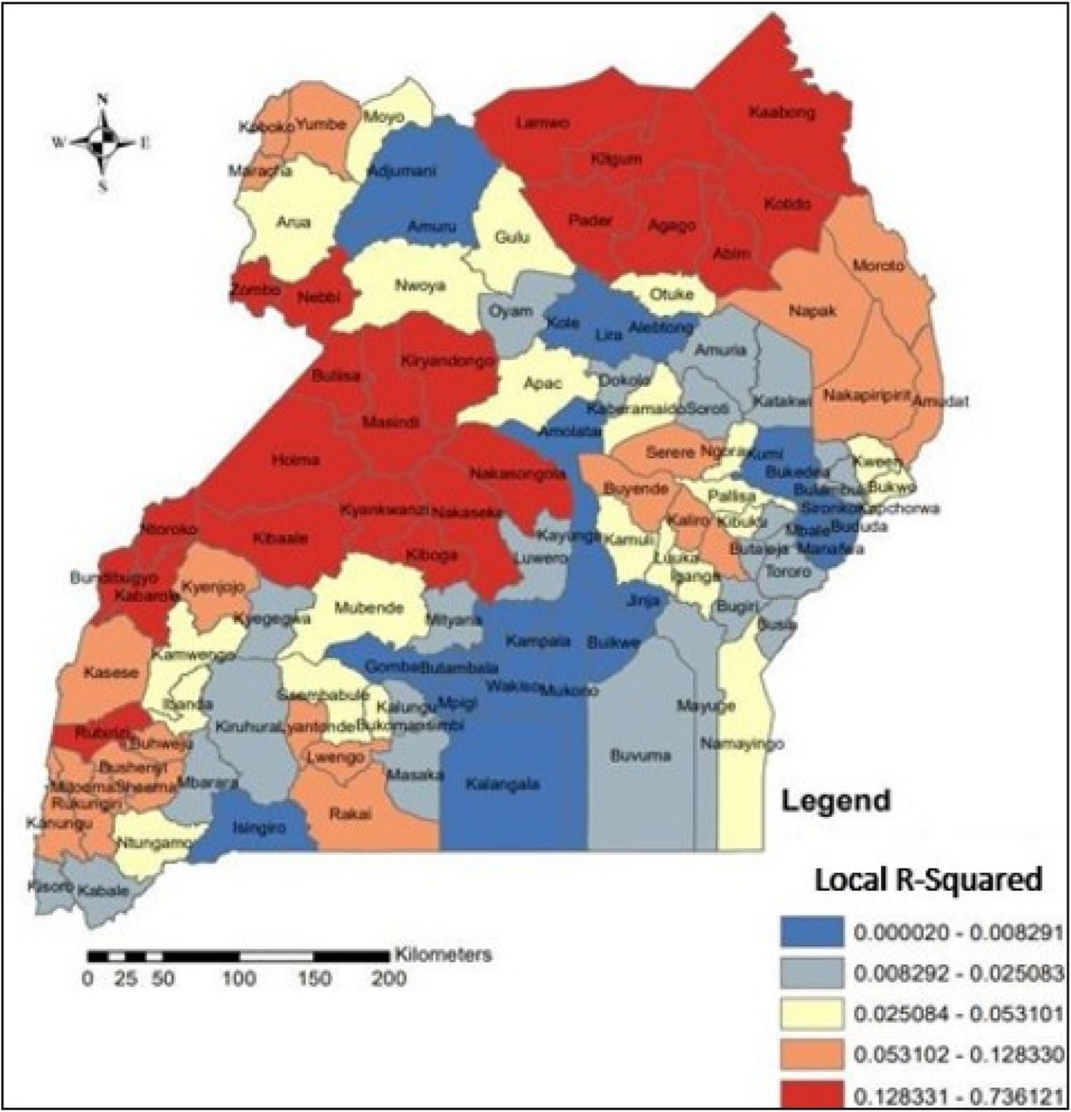
Geographically weighted correlation between Undernutrition of under five children and Poverty rates in Uganda

One of the aims of spatial analysis was to examine whether there was spatial autocorrelation between under-nutrition and its determinants across the country as well as to find hotspot or cold sport areas. Though the main focus was put on the correlation of undernutrition and its determinants, some attention was also given to analysis of the correlation within the determinants themselves (Fig. 2). Accordingly, the scatter graph in Fig. 2, shows that, the prevalence of undernutrition was positively correlated with poverty status (wealth quintile 1 and 2), percentage of children born with low birth weight, percentage of people born with low birth size, percentage of people with no toilet facilities and percentage of children whose delivery was outside health center. This implies that in general where these factors are high, undernutrition is also high. On the other hand, it was observed that, the prevalence of undernutrition was negatively correlated with; the proportion of parents who were able to read and write (literacy), percent of mothers who were working at the time of the survey, the percentage of household with access to water, percentage of children who had had vitamin A within the last six months preceding the survey and the percentage of single parents. Generally, where these factors were low, undernutrition was high and vice versa. Moreover, the plotted points in the scatter diagram represent points on the real ground for example taking into account the percentage of households within the first and second wealth quintiles “poor” and the percentage of children who were undernourished “undernutrition”, the correlation between the two variables was analyzed as indicated in Fig. 3 below. Figure 3 represents the spatially weighted coefficients of determinations (R-squared) for each location or district across the country.

**Fig. 4:**
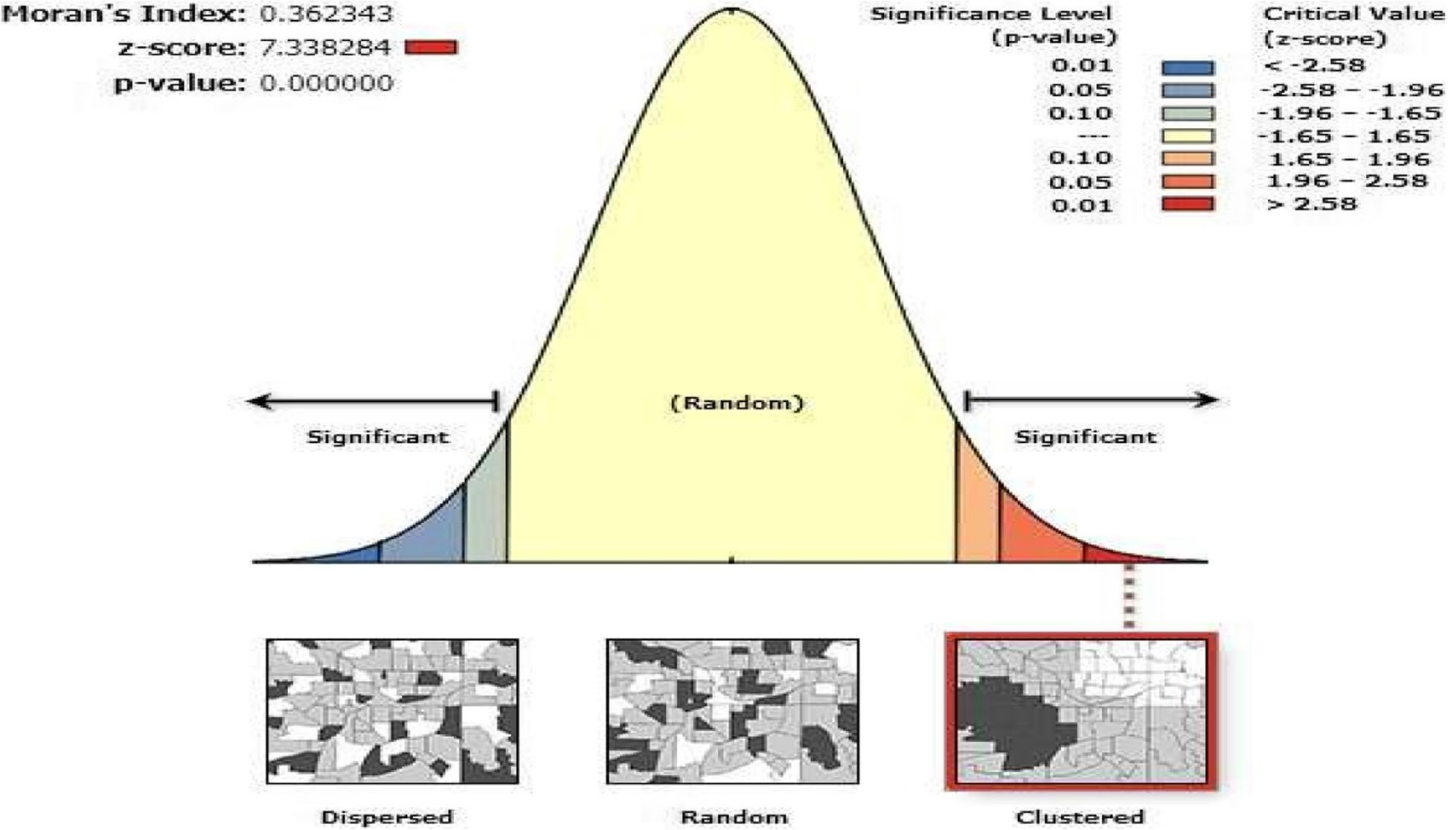
Moran's index of spatial autocorrelation for undernutrition in Uganda

Poverty and wealth are conceptually related and this said, the households within the lowest and second wealth quintiles are thus considered as poor. In this study therefore, it was apparent to consider and consequently use “poor” for those households within the first and second wealth quintiles. Observations made from Fig. 3 confirm the analysis displayed by the scatter diagram in Fig. 2 that, generally, areas in Uganda where the percentage of households within the first and second wealth quintiles was high, undernutrition was also high and generally, the areas that experienced low prevalence of undernutrition had lower proportions of poor households. A spatial autocorrelation index was generated to justify whether or not the overall autocorrelation of undernutrition or how closely the prevalence of undernutrition values are close to each other (Fig. 4).

**Fig. 5:**
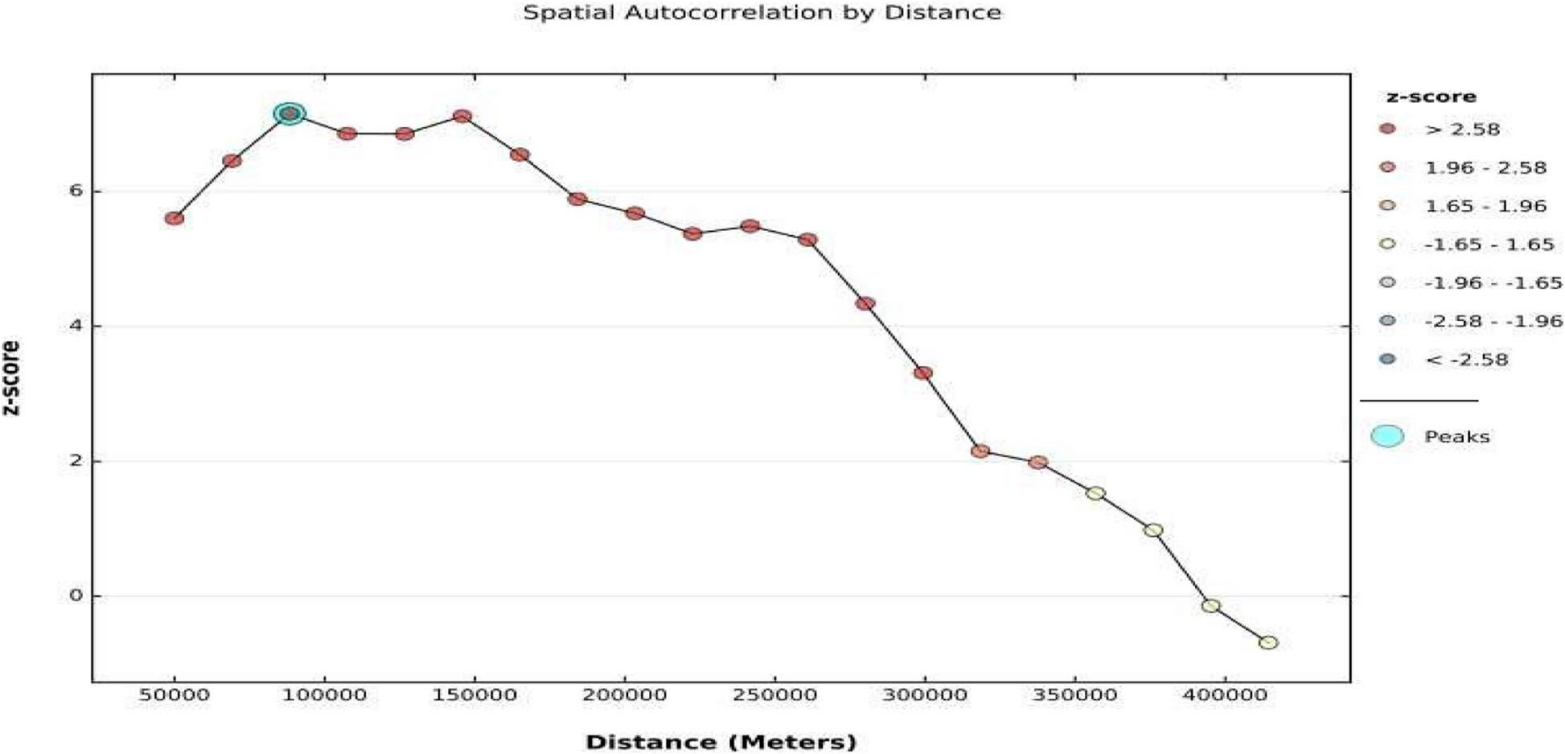
Global Moran's. Incremental spatial autocorrelation

More specifically, Moran’s I, an index for global spatial autocorrelation was used to measure the degree to which the prevalence of undernutrition was spatially different from the prevalence across the country. The analysis and the Moran’s I result, as indicated in Fig. 4 confirms clustering of the prevalence of undernutrition within the country and thus nullified the assumption of autocorrelation or random nature of the distribution of undernutrition. Observation made from Fig. 4, further indicate that the Moran’s I, index of spatial autocorrelation is positive 0.36 with a highly significant z-score of 7.3. Given the z-score value of 7.3, there is a less than 1% chance that this clustered pattern could be the result of random chance, but rather, there could be some influencing factors that strongly influence under-five children’s undernutrition some areas more than in some others. It was also important to examine the intensity of clustering distance varied. Global Moran’s I helped to examine spatial autocorrelation for a series of increasing distances as a way of measuring the intensity of spatial clustering as the distance varied (Fig. 5).

**Fig. 6:**
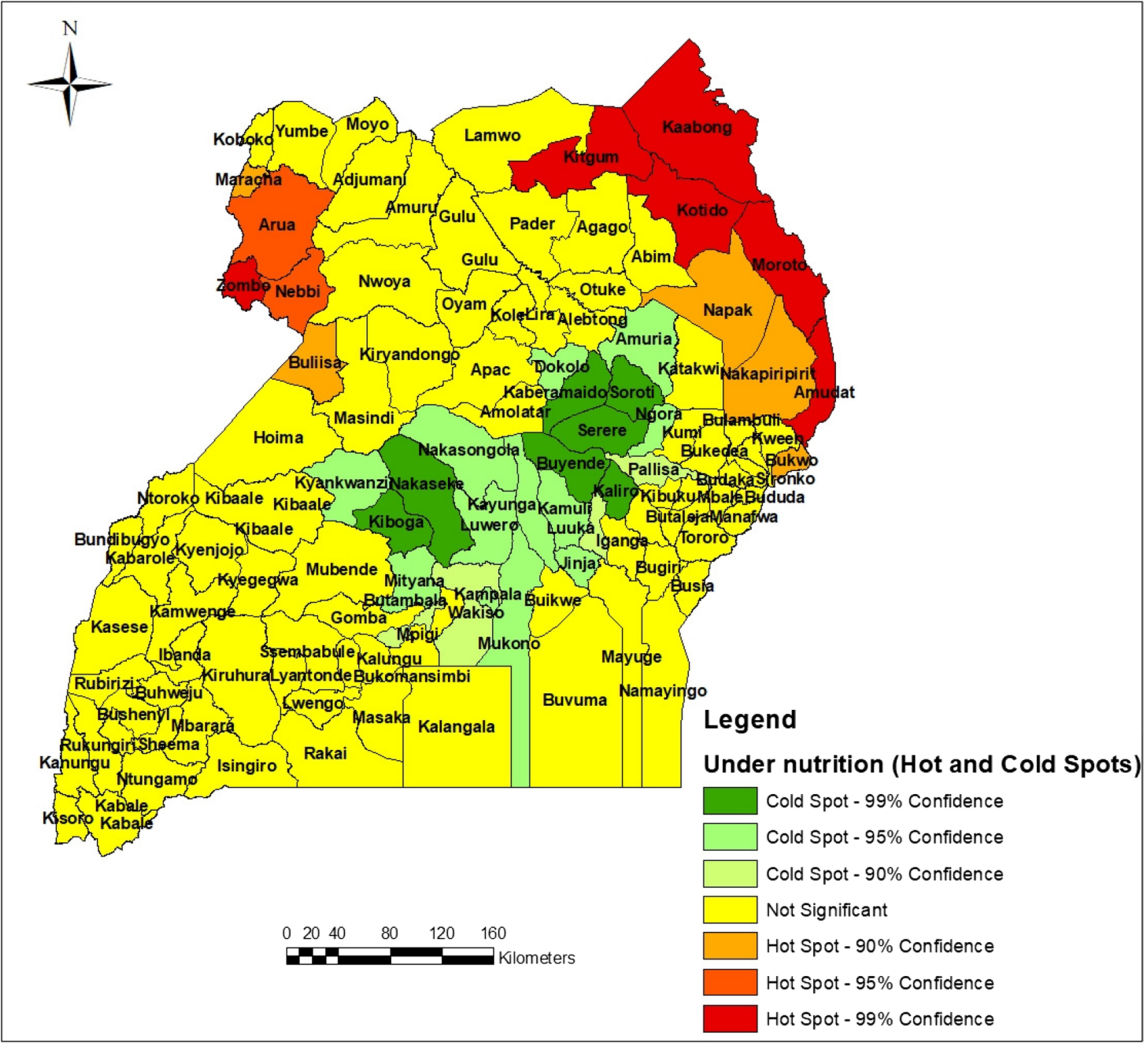
Distribution of undernutrition hot and cold spots in Uganda

Reference made to Fig. 5 shows that the peak at which clustering occurred is about 88 km. It was thus pertinent that after confirming the presence of autocorrelation and the confirmation of clustering of undernutrition, a hot spot analysis be carried out to identify the potential hotspot and or cold spot areas where clusters of undernutrition do exist. Accordingly, a GetisOrd Gi* tool was used to identify and locate the possibility and existence of hot spots of undernutrition as indicated in (Fig. 6).

**Fig. 7:**
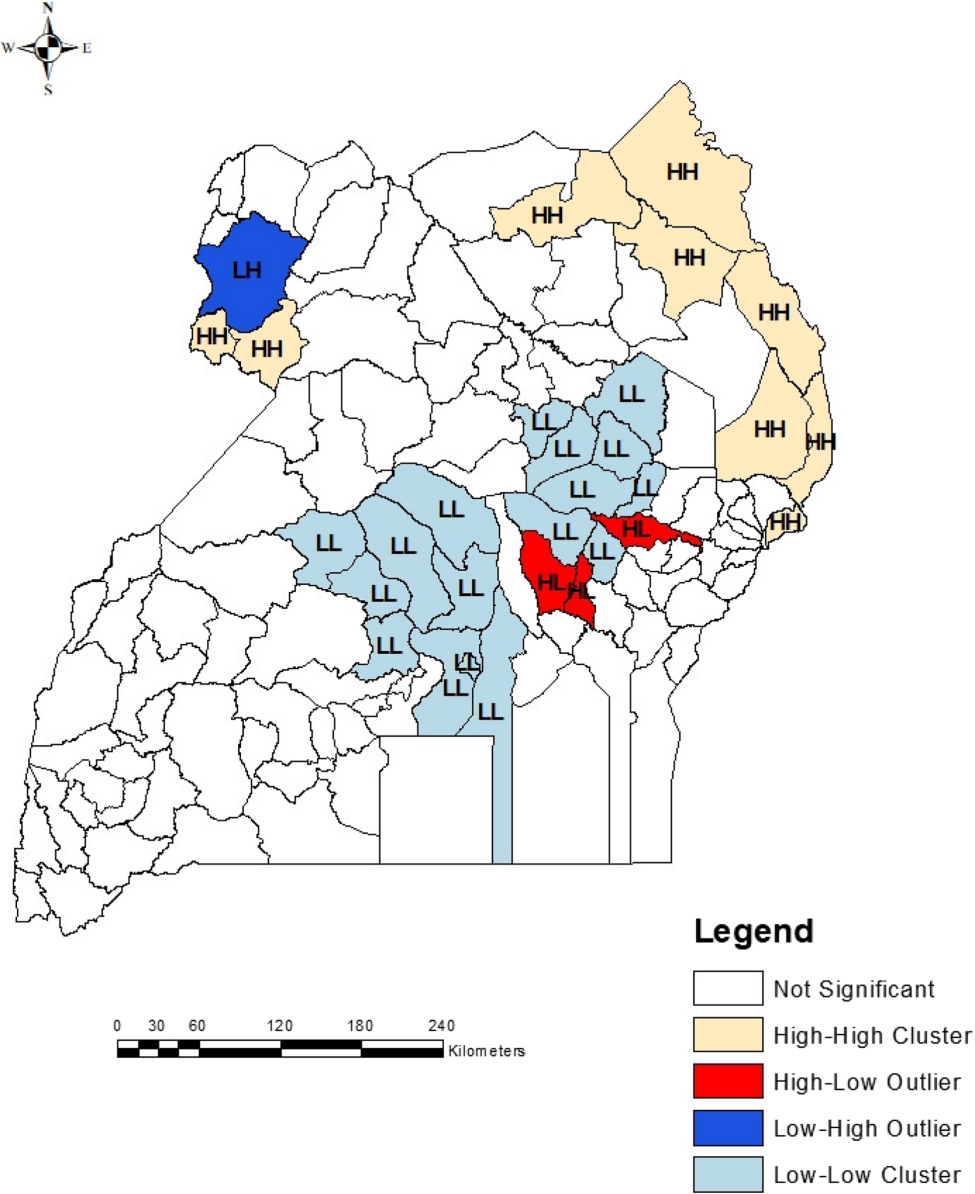
Distribution of outliers of the prevalence of under-five children undernutrition in Uganda

Accordingly, Fig. 6 indicates that, indeed undernutrition problem in Uganda is not random but rather clustered. Two potential hot spots and two potential cold spots were identified. The ArC GIS 10.7 tool for hotspot or cold spot analysis identified the potential hotspots with different levels of significance (confidence intervals of 90%, 95% and 99%) as can be seen from the map in Fig. 6. The potential cold spots were clustered around the central part of the country while the potential hotspot areas were clustered around the North Eastern (the Karamoja region), Bulisa and the North Western part (the West Nile region) of Uganda. Most parts of the country especially, the Western, South Western, Eastern and some parts of the North had no significant hot spots. There is normally a possibility of outliers existing within or around a hot spot or cold spot. Such scenarios are shown by existence of high value(s) of the attribute around a cold spot area or low value(s) of the attribute around a hotspot. The Anselin Local Moran’s I tool was used for this analysis as the results in Fig. 7 indicates.

**Fig. 8:**
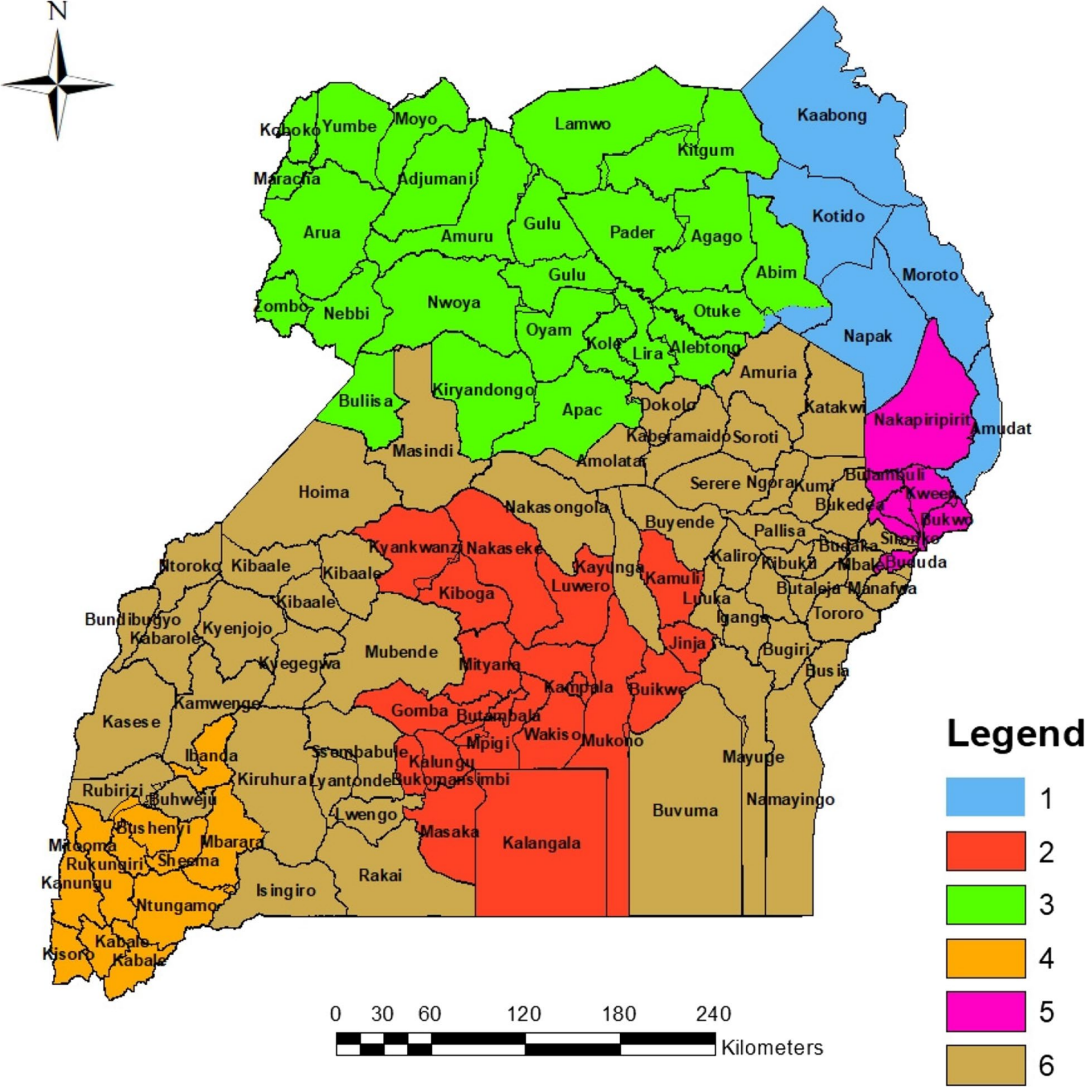
Grouping analysis of undernutrition and its determinants for children below five years of age in Uganda

**Fig. 9:**
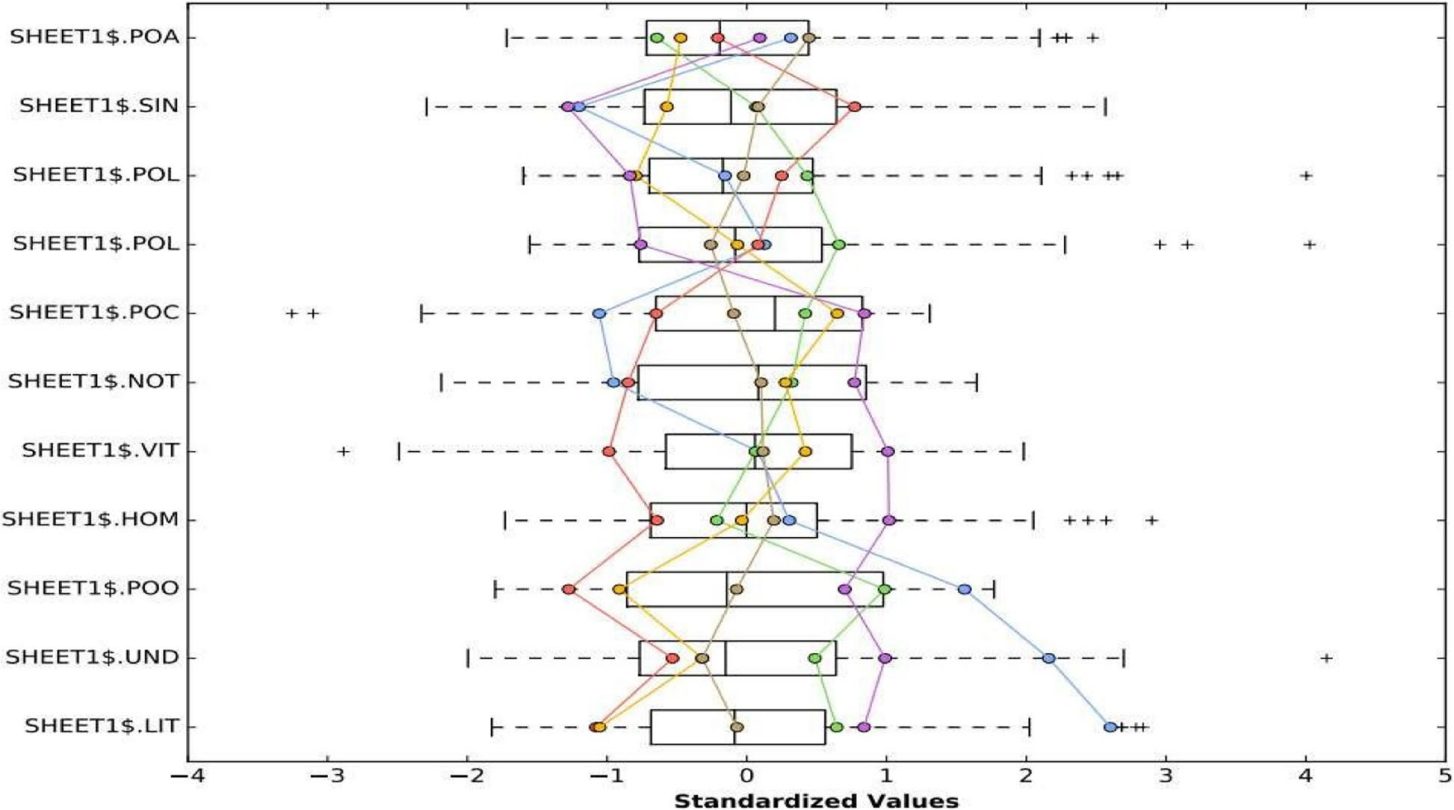
Grouping analysis for spatial variation of factor within different regions (clusters) whereby;Sheet1$.LIT (percentage of mother with ability to read and write), Sheet1$.UND (percentage of under-five children with undernutrition), Sheet1$.POO(percentage of households under wealth Quintile 1 and 2, Sheet1$.HOM(percentage of home deliveries), Sheet1$.VIT (percentage of under-five children who had taken vitamin A, Sheet1$.NOT (percentage of households with no toilet facilities), Sheet1$.POC (percentage of mothers were working at the time of the interviews), Sheet1$.POL (percentage of under-five children with low birth weight), Sheet1$.POL(percentage of under-five children with low birth size), Sheet1$.SIN( percentage of single mothers) and Sheet1$.POA (percentage of households with access to safe and clean water

Figure 7 shows much similarity between the hot spot and cold spot analysis reported in Fig. 6. The niche at this level of analysis was to identify whether the hotspot or cold spot areas were uniform within themselves or whether there were any possibilities of presence of outliers within and around them. From Fig. 7, it was observed that, some hotspots were surrounded with areas or districts with low undernutrition prevalence (HL), some cold spot areas were surrounded with areas with high prevalence (LH) of undernutrition while other hot or cold spots were homogenous (LL) or HH. It was also established that non-significant hot or cold spot areas (Fig. 6) remained nonsignificant with no significantly visible outliers (Fig. 7) within or in their neighbourhoods. Furthermore, in this study, it was prudent to identify the variations in determinants of undernutrition in each hot spot or cold spot area. With the help of grouping analysis clusters or groups were created such that within a given cluster, the attributes are as similar as possible and between different clusters, the attributes are as different as possible. The grouping analysis thus generated clusters with distinct and clear boundaries, where factors that had greater association amongst themselves were grouped together as portrayed in Figs. 8 and 9 below.

Six clusters were identified as distinguished groups of associations as indicated by the different colors in Fig. 8. The clusters generated were distinctly different from each other and the levels of association of each risk factor with undernutrition in each cluster was established as presented in Fig. 9. Group number 1 was basically the North Eastern region of the country (Karamoja region), a region where there is high prevalence of undernutrition according the findings presented earlier in Fig. 1. The second cluster was centered around Buganda region, the third was centered around the Northern region, the fourth was centered around the Ankole and Kigezi regions while the rest of the country was clustered in group 6 as seen in the Fig. 9. While in Fig. 8, the group are indicated by a number and color respectively, the same groups are indicated by colors in Fig. 9. In Fig. 9, each box the prevalence of each variable in different regions is shown as shown by the linkage of different colors on Figs. 8 and 9, for example the blue color in Fig. 9 corresponds to the blue color or group 1 on Fig. 8. As indicated in Fig. 9, the association between different variables indicated variations from group to group.

From Fig. 9, it was observed that within the North Eastern (the Karamoja region) as compared to the rest of the country, there existed; the highest level of illiteracy, the highest level of poverty (percentage of people within I and II wealth quintile), the highest levels of undernutrition, a bit lower than its neighbours in its south as far as home deliveries are concerned, average user of vitA. The region was also leading in having no toilet facilities in addition to having the lowest number of employed mothers, lowest number of single parents and has the highest percentage of people with no access to clean water. Earlier on, as indicated in Fig. 6, this zone was classified as a hot spot for undernutrition. It is therefore not surprising to find that the assumed risk factors were positively and significantly associated with undernutrition withing this very region of the country.

Furthermore, group 2 cluster which was basically within the central of the country and had been earlier classified as a cold spot of undernutrition (Fig. 6) was characterized by the lowest level of illiteracy, the lowest level of undernutrition, the lowest levels of poverty, the lowest level of home deliveries, lower levels of employed mothers and has the highest levels of single mothers with high percent of people with access to water. The Kigezi-Ankole cluster which was next to the central region cluster for most of the characteristics and of the attributes. The cluster was among those with the lowest levels of illiteracy, lower levels of undernutrition with prevalence just higher than the prevalence of the cluster in group two, levels of poverty just lower than those of cluster two, lower levels of single mothers, higher levels of working mothers and lower levels of people without access to clean water. The cluster in group 5 was characterized by among others; high levels of illiteracy, high levels of undernutrition, the highest levels of home delivery, the lowest levels of single mothers and higher levels people with no access to clean water. All these characteristics make clusters different from each other and it’s is important that before any one thinks of addressing and reducing the undernutrition problem in Uganda, understanding that different regions are affected differently and need different interventions is critical. As such it was necessary to examine the local significance of different risk factors in influencing undernutrition across the country. Consequently, Geographically Weighted Regression (GWR) model was generated as a way of visualizing the patterns of undernutrition and the possible influencing risk factors at local levels across the country.

### The spatial regression analysis of risk factors influencing undernutrition

**Fig. 10:**
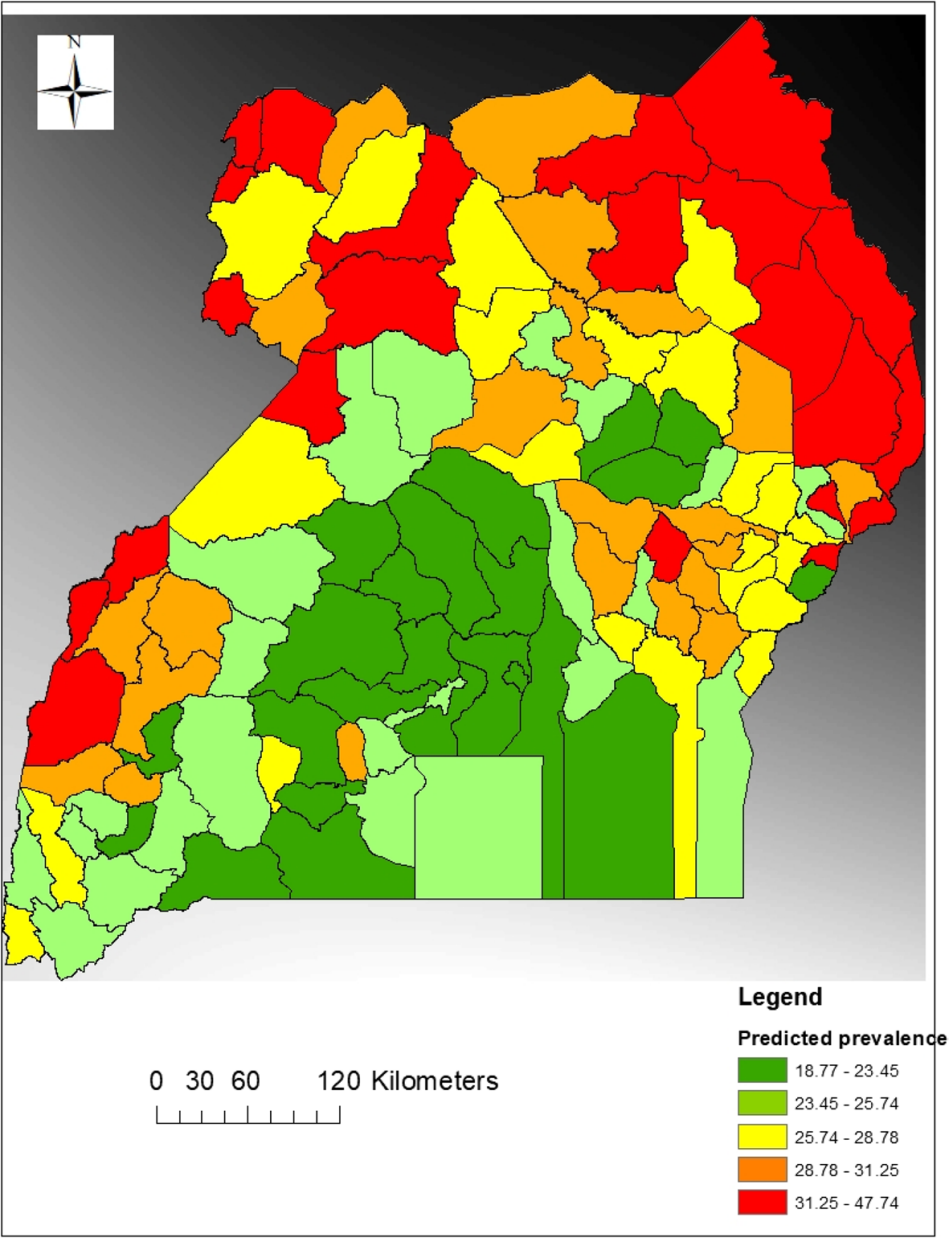
Geographically Weighted Regression: Spatial prediction of Undernutrition of under-five children in Uganda

Considering undernutrition as the dependent variable while observing while treating other variables as risk factors, the visual analysis outcome of the GWR as presented in Fig. 10, had great similarities with the autocorrelation or cluster analysis (Fig. 2) but more to this finding, it was established that the concentration of most risk factors as well as the outcome factors was within the North Eastern Uganda as indicated by the shadow intensity (Fig. 10).

The researchers, with the use of the GWR estimates were able to make predictions for undernutrition amongst under-five children in Uganda. The process of GWR generates a regression model for each location (each district) of the entire study. The output is thus a raster layer that corresponds to the spatial map generated (Fig. 10). As indicated in Fig. 10, much similarity between the patterns of actual values (Fig. 1) and predicted values (Fig. 10) is portrayed. Areas predicted to have high prevalence of undernutrition as predicted by the regression are close to the actual locations experiencing the real problem. This confirms therefore that GWR model can be used to predict undernutrition in Uganda and the fact that different areas are affected differently with different intensities of risk factors, the interventions to deal with the problem of undernutrition for under-five children in Uganda should be tailor made to the local level.

## Discussions

The findings of this study confirm that undernutrition amongst under-five children in Uganda was not randomly distributed. The prevalence of undernutrition varied by magnitude both within and between regions. Clustering of undernutrition prevalence was evident as well as significant existence of hot spots, cold spots and outliers as revealed by different analysis carried out. Spatial analysis for the risk factors which were believed to influence undernutrition amongst under-five children as per the literature reviewed also indicated spatial non-random distribution and existence of hotspots, cold spots and outliers alongside clusters of hot and cold spots areas (. The identification of hotspots through spatial analysis is an important element in designing measures for affected areas in a decentralized manner [12].

The distribution of undernutrition of under-five children in Uganda revealed fluctuation throughout the country with highest prevalence in the Toro region and the lowest prevalence within the Buganda region [6]. It was thus vital that the possibility of randomness in the nature of undernutrition and its influencers is scrutinized. The study was basically guided by spatial analysis approach and the tools of spatial analysis were used to justify various hypothesis.

Undernutrition being the main outcome variable was mapped using tools to identify the areas of greatest concern for under-five children Uganda. As indicated in Fig. 2, prevalence of undernutrition throughout the country was heterogenous suggesting spatial autocorrelation which was later confirmed by carrying out a spatial autocorrelation test. The spatial autocorrelation test as confirmed by Moran’s I confirmed the existence of global autocorrelation demystifying the possibility that the clustering is by chance. Although high prevalence of undernutrition was found across some selected districts through Uganda, clustering was higher in the regions of Karamoja, Buganda and West Nile. Studies done in India [9] revealed the elements of spatial heterogeneity in the distribution of undernutrition confirming clustering of undernutrition at district level and thus recommending that the approach should take into account clustering if the problem of undernutrition is to be solved.

The results of bivariate analysis were a mixture of positive, null and negative high or low correlations between the prevalence of undernutrition and the influencing factors. The current study reveals that districts with higher levels of poverty were associated with higher levels of undernutrition. Literature indicates that household wealth as was proxied by poverty in this study is strongly inversely correlated with wealth of nations and consequently, as many low- and middle-income countries achieve economic advancement and wealth, they pass through nutrition transition as such their rates decline [13].

Predictions and estimations for the prevalence of undernutrition generated using GWR indicated closeness between the observed and estimated prevalence of undernutrition amongst under-five children. Just like many other studies [14] the GWR analysis indicated spatial differences in the relative importance of undernutrition influencing factors. The intensity of influence of poverty and most other risk factors like lack of toilet facilities, literacy level, education level and distance to the health centers among others was stronger in the North Eastern Uganda than elsewhere. This finding suggests a localized approach to dealing with undernutrition challenge in Uganda.

Geographically weighted regression analysis indicated spatial differences in the relative importance of various poverty-influencing factors. Regions with high prevalence of poverty were associated with high intensity of undernutrition. This is no surprise as poverty and welfare of the households are highly associated in one way or the other. Poverty reinforces undernutrition by increasing the risk of food insecurity and likewise, undernutrition produces conditions of poverty by reducing the economic potential of the population [15]. The results also indicate that areas with high levels of illiteracy are associated with high intensity of undernutrition. Employment proportion of mothers with working status other than farm work was also associated with undernutrition. Elsewhere, education level and employment status of the household head were found as the main determinants of household welfare and poverty and thus children from such household are at a high risk of being malnourished [16].

The results of the GWR revealed high undernutrition rates in areas with; high illiteracy, high proportion of households within wealth indices i and ii, high percentage of low birth weight and perceived small size at birth as well as increased distance to the health centers. Communities with higher rates of home deliveries or deliveries that were not attended to by health professionals were also associated with higher prevalence rates.

## Conclusion

The current study reveals the fact that the distribution of undernutrition throughout the country is not uniform. Significant spatial patterns associated with undernutrition as were identified through hotspot and cold spot analysis existed in Uganda. Any programmes targeting to reduce the undernutrition amongst under-five children in Uganda should consider the spatial distribution and patterns of undernutrition and its determinants. Concentration of efforts to curb undernutrition amongst under-five children and prioritization of programmes should be given to the identified hotspot areas. The intensity of different risk factors of undernutrition amongst under-five children as revealed by the study should be given attention as this will guide on which factors to prioritize in improving the situation of under-five children in Uganda.

## Data Availability

The dataset that was analyzed to generate the can be accessed on request from the DHS programme via the DHS website (https://dhsprogram.com/data/dataset/Uganda_StandardDHS_2016.cfm?flag=1).
